# Pharmacokinetics of Piperaquine and Safety Profile of Dihydroartemisinin-Piperaquine Coadministered with Antiretroviral Therapy in Malaria-Uninfected HIV-Positive Malawian Adults

**DOI:** 10.1128/AAC.00634-18

**Published:** 2018-07-27

**Authors:** Clifford G. Banda, Fraction Dzinjalamala, Mavuto Mukaka, Jane Mallewa, Victor Maiden, Dianne J. Terlouw, David G. Lalloo, Saye H. Khoo, Victor Mwapasa

**Affiliations:** a Malawi College of Medicine, Blantyre, Malawi; b Malawi-Liverpool-Wellcome Trust Clinical Research Programme, Blantyre, Malawi; c Oxford Centre for Tropical Medicine and Global Health, Oxford, United Kingdom; d Liverpool School of Tropical Medicine, Liverpool, United Kingdom; e University of Liverpool, Liverpool, United Kingdom; f Mahidol-Oxford Tropical Medicine Research Unit, Bangkok, Thailand

**Keywords:** piperaquine, antiretroviral therapy, malaria, antiretroviral agents

## Abstract

There are limited data on the pharmacokinetic and safety profiles of dihydroartemisinin-piperaquine (DHA-PQ) among human immunodeficiency virus-infected (HIV-positive [HIV^+^]) individuals taking antiretroviral therapy (ART). In a two-step (parallel-group) pharmacokinetic trial with intensive blood sampling, we compared the area under the concentration-time curve from days 0 to 28 (AUC_0–28 days_) and the safety outcomes of piperaquine among malaria-uninfected HIV^+^ adults. In step 1, half the adult dose of DHA-PQ was administered for 3 days as an initial safety check to four groups (*n* = 6/group) of HIV^+^ adults (age ≥18 years): (i) antiretroviral-naive individuals, (ii) individuals on nevirapine-based ART, (iii) individuals on efavirenz-based ART, and (iv) individuals on ritonavir-boosted lopinavir-based ART. In step 2, a full adult treatment course of DHA-PQ was administered to a different cohort of participants in three groups: (i) antiretroviral-naive individuals, (ii) individuals on efavirenz-based ART, and (iii) individuals on nevirapine-based ART (*n* = 10 to 15/group). The ritonavir-boosted lopinavir-based ART group was dropped in step 2 due to the limited number of participants who were on this second-line ART and were eligible for recruitment. Piperaquine's AUC_0–28 days_ in both steps was 43% lower among participants on efavirenz-based ART than among ART-naive participants. There were no significant differences in AUC_0–28 days_ between the other ART groups and the ART-naive group in each of the two steps. Furthermore, no differences in treatment-emergent clinical and laboratory adverse events were observed across the groups in steps 1 and 2. Although it was well tolerated at the half and full standard adult treatment courses, the efavirenz-based antiretroviral regimen was associated with reduced piperaquine exposure, which may compromise dihydroartemisinin-piperaquine's effectiveness in programmatic settings. (The clinical trials presented in this study have been registered at the WHO's International Clinical Trials Registry Platform under ID numbers PACTR2010030001871293 and PACTR2010030001971409.)

## Abstract (precis)

There are limited data on the pharmacokinetic and safety profiles of dihydroartemisinin-piperaquine (DHA-PQ) among human immunodeficiency virus-infected (HIV-positive [HIV^+^]) individuals taking antiretroviral therapy (ART). In a two-step (parallel-group) pharmacokinetic trial with intensive blood sampling, we compared the area under the concentration-time curve from days 0 to 28 (AUC_0–28 days_) and the safety outcomes of piperaquine among malaria-uninfected HIV^+^ adults.

## INTRODUCTION

Human immunodeficiency virus (HIV) and Plasmodium falciparum malaria infections are endemic in most areas in sub-Saharan Africa (SSA), and coinfections occur frequently. HIV infection increases susceptibility to malaria (1, 2) and the severity of P. falciparum malaria (3–6) and reduces the efficacy of some antimalarial drugs in current use (7, 8). To combat these dual infections, the World Health Organization (WHO) recommends initiation of antiretroviral therapy (ART) in HIV-positive (HIV^+^) individuals and prompt use of artemisinin-based combination therapies (ACTs). Dihydroartemisinin (DHA)-piperaquine (PQ) is one of the ACTs increasingly being used in SSA in malaria-infected individuals (9) owing to its better safety profile and longer piperaquine half-life of approximately 33 days (10, 11), which make it an ideal option for the treatment of uncomplicated P. falciparum malaria (12, 13) and the intermittent preventive treatment of malaria in pregnancy (14, 15). Additionally, dihydroartemisinin, which has a half-life of approximately 1 h, is fast acting, and is 5 to 10 times more potent than the other artemisinin derivatives (16). Because of the geographical overlap of malaria and HIV infection, DHA-PQ will likely be commonly coadministered with ART, such as efavirenz (EFV), nevirapine (NVP), or ritonavir-boosted lopinavir (LPV/r).

It has been postulated that pharmacokinetic (PK) interactions between ACTs and nonnucleoside reverse transcriptase inhibitor (NNRTI)- or protease inhibitor (PI)-containing ART are likely since these classes of drugs affect the activity of cytochrome P450 (CYP450) liver enzymes. NNRTIs, such as NVP and EFV, usually induce various CYP450 isoforms, but they are also substrates for CYP450 enzymes, as are ACTs. Conversely, HIV PIs, particularly ritonavir, are potent inhibitors of CYP3A enzymes (17), which form part of the CYP450 enzyme entity. Administration of ACTs in HIV^+^ individuals on ART may therefore reduce or increase the plasma concentrations of any of the drug components of ACTs. Dihydroartemisinin may have limited pharmacokinetic interactions with ART since it is metabolized through glucuronidation by UDP glucuronosyltransferase (18). However, piperaquine, as a xenobiotic, is metabolized by CYP450 (CYP3A4 and CYP2C8) for excretion (19). Any induction or inhibition of these enzymes by ART may affect the clearance of piperaquine and, therefore, its efficacy and safety.

In a two-step (parallel), intensive pharmacokinetic sampling trial, we compared the safety of DHA-PQ and secondary pharmacokinetic parameters (area under the concentration-time curve [AUC] from days 0 to 28 [AUC_0–28 days_], maximum concentration [*C*_max_], the time to the maximum concentration [*t*_max_], elimination half-life [*t*_1/2_]) of piperaquine between HIV^+^ adults taking various ART (efavirenz-, nevirapine-, or ritonavir-boosted lopinavir-based regimens) and HIV^+^ adults not on any ART.

## RESULTS

### Characteristics of study participants.

In step 1, 24 participants (6 in each group) were enrolled and successfully followed up for 28 days; these participants included 5 who replaced those withdrawn due to protocol violations. In step 2, 40 participants were enrolled (10 in the ART-naive group and 15 in each of the EFV and NVP groups) and completed 28 days of follow-up; these participants included 2 who replaced those withdrawn due to protocol violations. In accordance with the protocol, data for withdrawn individuals were not included in the PK analyses. As shown in Table 1, participants who completed the follow-up in steps 1 and 2 generally had similar baseline characteristics. In step 1, those on ritonavir-boosted lopinavir had a longer median duration of ART intake than those on EFV and NVP. In addition, the baseline alanine aminotransferase (ALT) concentration was higher in those on EFV-based ART.

**TABLE 1:** Baseline characteristics for study participants in step 1 and step 2

Characteristic	Step 1					Step 2			
	Value(s) for participants receiving:				*P* value	Value(s) for participants receiving:			*P* value
	DHA-PPQ + NVP-containing ART (*n* = 5)	DHA-PPQ + EFV-containing ART (*n* = 6)	DHA-PPQ + LPV/r-containing ART (*n* = 6)	DHA-PPQ without ART (*n* = 6)		DHA-PPQ + NVP-containing ART (*n* = 15)	DHA-PPQ + EFV-containing ART (*n* = 15)	DHA-PPQ without ART (*n* = 10)	
No. (%) of female participants	3 (50.0)	2 (33.3)	2 (33.3)	4 (66.7)	0.811	13 (86.7)	13 (86.7)	5 (50.0)	0.071
Median (range) age (yr)	39 (34–62)	43 (36–56)	41 (20–63)	29 (23–46)	0.360	36 (28–44)	36 (24–60)	40 (33–62)	0.060
Mean (SD) hemoglobin concn (g/dl)	13.9 (1.3)	12.7 (1.6)	13.1 (1.6)	12.9 (1.0)	0.633	13.3 (2.1)	13.4 (2.2)	13.9 (2.9)	0.830
Median (range) body mass index (kg/m^2^)	24.3 (22.0–25.5)	20.4 (18.7–23.1)	19.8 (17.5–25.7)	23.9 (19.9–26.4)	0.071	23.1 (18.0–28.8)	20.9 (16.0–19.0)	21.3 (18.4–27.4)	0.602
Median (range) duration of ART intake at time of screening (mo)	26.3 (7.0–55.7)	24.5 (15.2–49.9)	65.7 (52.2–86.9)	NA^*a*^	0.020	47.7 (10.2–80.4)	39.8 (7.1–120.1)	NA	0.371
No. (%) of participants on co-trimoxazole prophylaxis	6 (100.0)	6 (100.0)	6 (100.0)	6 (100.0)	1.000	13 (86.7)	13 (86.7)	7 (70.0)	0.511
Median (range) ALT concn (IU/liter)	26 (12–39)	35 (20–44)	20 (15–23)	18 (11–19)	0.024	23 (15–39)	22 (11–38)	21 (17–28)	0.750
No. (%) of participants with AST >ULN^*b*^	2 (33.3)	4 (66.7)	0 (0.0)	0 (0.0)	0.092	5 (33.3)	3 (20.0)	3 (30.0)	0.743
Median (range) AST concn (IU/liter)	27 (19–58)	39 (24–46)	29.5 (21–35)	23 (19–27)	0.081	27 (17–52)	29 (21–53)	28 (20–34)	0.524
No. (%) of participants with ALT >ULN	2 (33.3)	3 (50.0)	0 (0.0)	0 (0.0)	0.284	3 (20.0)	3 (20.0)	2 (20.0)	1.000
Median (range) creatinine concn (μmol/liter)	67 (42–139)	57 (38–67)	73 (44–90)	58 (51–69)	0.332	60 (41–83)	55 (32–69)	59 (47–68)	0.871
No. (%) of participants with creatinine concn >ULN	2 (33.3)	0 (0.0)	0 (0.0)	0 (0.0)	0.221	0 (0.0)	0 (0.0)	0 (0.0)	1.000
No. (%) of participants with:									
Any anemia	0 (0.0)	0 (0.0)	0 (0.0)	0 (0.0)	1.000	2 (22.0)	0 (0.0)	0 (0.0)	0.330
Any leucopenia	0 (0.0)	0 (0.0)	0 (0.0)	1 (16.7)	1.000	5 (33.3)	1 (6.7)	3 (30.0)	0.232
Any neutropenia	2 (33.3)	2 (33.3)	4 (66.7)	1 (16.7)	0.460	3 (20.0)	5 (33.3)	3 (30.0)	0.741
Any thrombocytopenia	0 (0.0)	0 (0.0)	0 (0.0)	1 (16.7)	1.000	4 (26.7)	1 (6.7)	1 (10.0)	0.410
Median (range) CD4 cell count (no. of cells/μl)	441 (254–832)	386 (273–757)	422 (375–691)	411 (324–734)	0.670	476 (298–685)	389 (274–1,222)	429 (393–888)	0.311

a NA, not applicable.

b >ULN, greater than the upper limit of normal.

### Pharmacokinetic interactions between piperaquine and ART in step 1.

**FIG 1:**
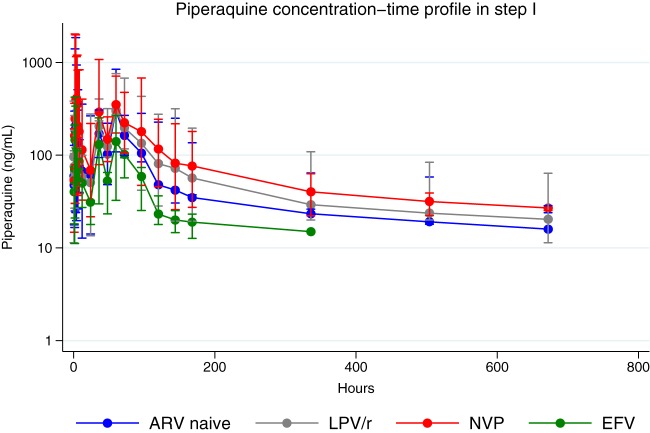
Piperaquine concentration-time profile (semilogarithmic scale) following administration of half of the standard dihydroartemisinin-piperaquine adult dose in step 1 (*n* = 23 [the data for one participant were excluded]; ART-naive individuals, *n* = 6; individuals receiving efavirenz [EFV], *n* = 6; individuals receiving ritonavir-boosted lopinavir [LPV/r], *n* = 6; individuals receiving nevirapine [NVP], *n* = 5). Concentrations below the lower limit of quantification were excluded, resulting in a plotted observation time of up to 336 h in the efavirenz group and 672 h in the rest of the study groups. Data are represented as the mean (95% confidence interval).

Participants in the EFV-ART group had 43% lower AUC_0–28 days_ of piperaquine than those in the ART-naive group (geometric mean ratio, 0.57 [90% confidence interval {CI}, 0.38 to 0.83]; *P* = 0.029). There were no significant differences in AUC_0–28 days_ among participants in the other ART groups from those among participants in the ART-naive group. Piperaquine's *C*_max_ was higher in the NVP-ART group than in the ART-naive group (geometric mean ratio, 1.82 [90% CI, 1.13 to 2.94]; *P* = 0.061), but no significant differences in *C*_max_ were observed between the rest of the ART groups and the ART-naive group. There were no significant differences in the *t*_1/2_ of piperaquine in all four study groups (as shown in Table 2). However, the median *t*_max_ was higher in the LPV–r-ART group than in the ART-naive group (*P* = 0.049). Figure 1 shows the concentration-time profile between ART groups and the ART-naive group. There was a lower piperaquine concentration-time profile in the EFV-ART group than in the ARV-naive group.

**TABLE 2:** Piperaquine pharmacokinetic parameters for participants in step 1^*a*^

PK parameter	Values^*b*^ for participants in the following study groups:				NVP/ART-naive participants		LPV/r/ART–naive participants		EFV/ART-naive participants	
	ART naive (*n* = 6)	NVP (*n* = 5)^*c*^	LPV/r (*n* = 6)	EFV (*n* = 6)	Geometric mean ratio (90% CI)	*P* value^*d*^	Geometric mean ratio (90% CI)	*P* value	Geometric mean ratio (90% CI)	*P* value
AUC_0–28 days_ (ng · h/ml)	33,385 (26,131–42,652)	43,632 (31,383–60,662)	38,300 (27,256–53,802)	18,914 (14,144–25,291)	1.31 (0.86–1.99)	0.290	1.15 (0.75–1.76)	0.589	0.57 (0.38–0.83)	0.029
*C*_max_ (ng/ml)	350 (252–485)	637 (453–897)	327 (263–406)	253 (156–412)	1.82 (1.13–2.94)	0.061	0.94 (0.63–1.39)	0.775	0.72 (0.40–1.32)	0.371
*t*_max_ (h)	3 (2–60)	4 (3–5)	60 (60–60)	3 (2–60)		0.573^*e*^		0.049^*e*^		1.000^*e*^
*t*_1/2_ (h)^*f*^	332 (174–631)	319 (262–388)	455 (186–1,114)	227 (120–432)	0.36 (0.49–1.89)	0.915	1.37 (0.44–4.31)	0.636	0.68 (0.36–1.30)	0.658

a ART, antiretroviral therapy; NVP, nevirapine-based ART; EFV, efavirenz-based ART; LPV/r, ritonavir-boosted lopinavir-based ART; *C*_max_, maximum concentration, *t*_max_, the time to reach the maximum concentration, *t*_1/2_, drug elimination half-life; AUC_0–28 days_, area under the concentration-time curve from days 0 to 28; *C*_d7_, day 7 piperaquine concentration.

b Values are presented as the geometric mean (90% confidence interval) for all PK parameters except *t*_max_, the values of which are given as the median (interquartile range).

c One participant did not complete follow-up and was excluded from the analysis.

d *P* values were calculated using analysis of variance (ANOVA) in STATA (version 15.0) (α = 0.1), unless indicated otherwise.

e This *P* value was calculated using the Wilcoxon rank-sum test (α = 0.05).

f Half-life estimation excluded values below the lower limit of quantification for each participant.

### Safety assessment in step 1.

DHA-PQ was well tolerated in all study groups. However, one participant in the ART-naive group had a 3-day history of headache, heart palpitations, nausea with no vomiting, and good appetite following the intake of DHA-PQ. These resolved by day 7 of follow-up. One participant in the NVP-ART group developed left-sided hemiplegia which was not thought to be associated with the coadministration with DHA-PQ. There were no clinically significant treatment-emergent hematological or hepatic abnormalities across the study groups.

### Pharmacokinetic interactions between piperaquine and ART in step 2.

**FIG 2:**
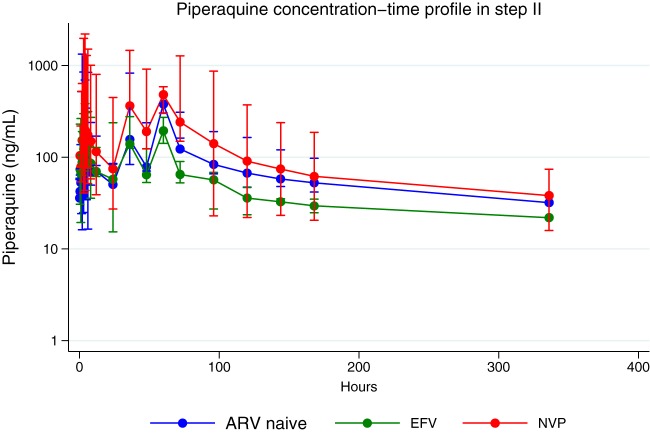
Piperaquine concentration-time profile (semilogarithmic scale) following administration of a full standard adult dose of dihydroartemisinin-piperaquine in step 2 (*n* = 40; ART-naive individuals, *n* = 10; individuals receiving efavirenz [EFV], *n* = 15; individuals receiving nevirapine [NVP], *n* = 15). Concentrations below the lower limit of quantification were excluded, resulting in a plotted observation time up to 336 h. Data are represented as the mean (95% confidence interval).

In step 2, piperaquine's AUC_0–28 days_ was 43% lower in the EFV-ART group than in the ART-naive group (geometric mean ratio, 0.57 [95% CI, 0.44 to 0.74]; *P* = 0.002). There was no significant difference in piperaquine's AUC_0–28 days_ between the NVP-ART and ART-naive groups. Furthermore, participants in the EFV-ART group had a 43% lower *C*_max_ of piperaquine than the ART-naive group (geometric mean ratio, 0.57 [95% CI, 0.36 to 0.90]; *P* = 0.065), and piperaquine's *t*_1/2_ was 64% lower in the EFV-ART group than in the ART-naive group (geometric mean ratio, 0.36 [95% CI, 0.15 to 0.87]; *P* = 0.072). However, there were no significant differences in the *C*_max_ and *t*_1/2_ of piperaquine between the NVP-ART and the ART-naive groups, as shown in Table 3. Similarly, no significant differences in the median *t*_max_ between the two ART groups and the ART-naive group were observed. Figure 2 illustrates the piperaquine concentration-versus-time plot in the NVP, EFV, and ART-naive groups in step 2. The EFV-ART group had a lower concentration-time profile of piperaquine than the ART-naive group, and there was a tendency toward higher piperaquine concentrations in the NVP-ART group than in the ART-naive group.

**TABLE 3:** Piperaquine pharmacokinetic parameters for participants in step 2^*a*^

PK parameter	Values^*b*^ for participants in the following study groups			NVP/ART-naive participants		EFV/ART-naive participants	
	ART naive (*n* = 10)	NVP (*n* = 15)^*c*^	EFV (*n* = 15)	Geometric mean ratio (90% CI)	*P* value^*d*^	Geometric mean ratio (90% CI)	*P* value
AUC_0–28 days_ (ng · h/ml)	27,573 (23,208–32,759)	36,747 (28,419–47,516)	15,792 (13,094–19,048)	1.33 (0.98–1.82)	0.179	0.57 (0.44–0.74)	0.002
*C*_max_ (ng/ml)	430 (315–587)	557 (424–731)	245 (175–343)	1.30 (0.85–1.96)	0.314	0.57 (0.36–0.90)	0.065
*t*_max_ (h)	60 (60–60)	60 (36–60)	60 (24–60)		0.841^*e*^		0.441^*e*^
*t*_1/2_ (h)^*f*^	136 (72–255)	76 (36–160)	49 (27–90)	0.56 (0.21–1.51)	0.356	0.36 (0.15–0.87)	0.072
*C*_d7_ (ng/ml)^*g*^	53 (39–71)	62 (46–84)	39 (32–48)	1.17 (0.76–1.83)	0.519	0.74 (0.51–1.07)	0.469

a ART, antiretroviral therapy; NVP, nevirapine-based ART; EFV, efavirenz-based ART; LPV/r, ritonavir-boosted lopinavir-based ART; *C*_max_, maximum concentration, *t*_max_, the time to reach the maximum concentration, *t*_1/2_, drug elimination half-life; AUC_0–28 days_, area under the concentration-time curve from days 0 to 28; *C*_d7_, day 7 piperaquine concentration.

b Values are presented as the geometric mean (90% confidence interval) for all PK parameters except *t*_max_, the values of which are given as the median (interquartile range).

c One participant did not complete follow-up and was excluded from the analysis.

d *P* values were calculated using analysis of variance (ANOVA) in STATA (version 15.0) (α = 0.1), unless indicated otherwise.

e This *P* value was calculated using the Wilcoxon rank-sum test (α = 0.05).

f Half-life estimation excluded values below the lower limit of quantification for each participant.

g Values below the lower limit of quantification were excluded, resulting in the following number of observations (*n* = 22): for the ART-naive group, *n* = 2; for the NVP group, *n* = 10; and for the EFV group, *n* = 10.

### Piperaquine day 7 concentrations.

Of the 40 participants in step 2, 22 had piperaquine plasma concentration above the lower limit of quantification (>25 ng/ml) at day 7 posttreatment. There was no evidence of a significant difference in the day 7 piperaquine concentration across the ART groups (Table 3). Of the 22 participants with a day 7 piperaquine concentration above >25 ng/ml (ART-naive group, *n* = 2; EFV ART group, *n* = 10; NVP ART group, *n* = 10), the proportion achieving piperaquine concentrations of >30 ng/ml was 90% (*n* = 10) in the ART-naive group, 100% (*n* = 2) in the EFV ART group, and 90% (*n* = 10) in the NVP ART group. There was no evidence of a difference in these proportions between each of the EFV and NVP ART groups and the ART-naive group (for EFV and NVP ART versus the ART-naive group, *P* = 1.000 for both comparisons).

### Safety assessment in step 2.

DHA-PQ was generally well tolerated in all study groups in step 2. However, one participant in the ART-naive group reported nausea following intake of DHA-PQ, but this resolved within a day. The proportions of study participants who had any grade of treatment-emergent transaminitis (elevated ALT and aspartate aminotransferase [AST] levels) after DHA-PQ administration were similar in the ART-naive and EFV ART groups (50% [5/10] versus 40% [6/15], respectively; *P* = 0.697) and between the ART-naive and NVP ART (53% [8/15]) groups (*P* = 1.000). None of the elevated AST or ALT levels reached severity levels of grade 3 or 4 or were persistent beyond day 28 of follow-up. The proportions of participants who had any grade of treatment-emergent neutropenia after DHA-PQ administration were similar between the ART-naive (30% [3/10]) and the EFV ART (33% [5/15]) groups (*P* = 1.000) and between the ART-naive and the NVP ART (20% [3/15]) groups (*P* = 0.653). There were no cases reaching grade 3 or 4 neutropenia in any of the groups. Additionally, the proportions of participants who had a QTc prolongation after DHA-PQ administration (470 ms at day 3 of follow-up) were 0.0% (0/10), 13.3% (2/15), and 13.3% (2/15) in the ART-naive, EFV ART, and NVP ART groups, respectively, with no evidence of a significant difference between the NVP and EFV ART groups and the ART-naive group being detected. All cases of QTc prolongation resolved spontaneously by day 21 of follow-up.

### Dose proportionality between ART-naive participants in steps 1 and 2.

Assuming a linear disposition of piperaquine, increasing the dose in step 2 should result in an increased AUC_0–28 days_ in this step compared to that in step 1. As part of an exploratory analysis, not determined *a priori*, we assessed dose proportionality between the ART-naive groups in steps 1 and 2 using a linear quadratic regression approach by regressing dose-normalized AUC_0–28 days_ (AUC_0–28 days_/dose) with the total dose received by each participant (20). The fitted linear regression equation was

(1) $${\text{AUC}}_{0-28\;\;\;\text{days}}/\text{dose}=\alpha +\beta_{1}\cdot \text{dose}+\beta_{2}\cdot {\text{dose}}^{2}$$

The null hypothesis was that β_2_ and α coefficients are equal to zero. Dose proportionality was declared if α and β_2_ were not significantly different from zero. Equation 1 could be further simplified to equation 3 when β_2_ is not significantly different from zero:

(2) $${\text{AUC}}_{0-28\;\;\;\text{days}}/\text{dose}=\alpha +\beta \cdot \text{dose}$$

Neither equation showed evidence against the null hypothesis, as illustrated below in the result of equation 1 which was derived from ART-naive participants in steps 1 and 2, showing that β_2_ and α were not very significantly different from zero:

$${\text{AUC}}_{0-28\;\;\;\text{days}}/\text{dose}=0.116-0.00011\cdot \text{dose}+3.37\text{e}-08\cdot {\text{dose}}^{2}$$

## DISCUSSION

The aim of this study was to compare the secondary pharmacokinetic parameters of piperaquine and the safety of dihydroartemisinin-piperaquine between HIV-infected adults taking various antiretroviral therapies (efavirenz-, nevirapine-, and ritonavir-boosted lopinavir-based regimens) and HIV-infected adults not on any antiretroviral therapy. We found that the coadministration of piperaquine and the efavirenz-based ART regimen significantly lowered piperaquine's exposure (AUC_0–28 days_) at half and full standard adult courses, reduced piperaquine's half-life, and achieved maximum concentration at full standard adult course compared with the values obtained when piperaquine was administered alone among non-malaria HIV-infected adults. Additionally, the day 7 piperaquine concentration was not significantly different between the ART groups following intake of a full standard adult course. Furthermore, DHA-PQ was well tolerated at both half and full standard adult courses across all ART groups, with no evidence of significant differences in treatment-emergent clinical and laboratory adverse events across all ART groups.

The finding of a significantly lower piperaquine concentration in the EFV group in both steps is consistent with the known metabolism of EFV, which is a potent inducer of CYP3A4 (17) and is one of the major CYP450 isoforms responsible for the metabolic clearance of piperaquine (19). There is paucity of published evidence on the interaction between piperaquine and ART among nonpregnant individuals. However, our findings are consistent with previous findings among pregnant women receiving DHA-PQ for intermittent preventive treatment against malaria in Uganda, where piperaquine exposure was shown to be 38% lower among pregnant women receiving EFV-based ART than among HIV-uninfected pregnant women (21). Thus, in the present study, EFV induction of CYP3A4 in the EFV-treated group might have led to the enhanced clearance and shorter half-life of piperaquine seen in step 2.

Unexpectedly, we found a nonsignificantly higher concentration of piperaquine in the NVP-based ART group in steps 1 and 2 than in the ART-naive group. While there is some evidence that NVP induces CYP3A4 (22, 23), other studies have suggested that it may act as an inhibitor of other drugs metabolized by CYP3A4, as shown by the increased *C*_max_ and AUC of darunavir (24) and maraviroc (25) when coadministered with NVP. The nonsignificantly increased AUC_0–28 days_ and *C*_max_ of piperaquine in our study could suggest increased bioavailability or reduced metabolism. As this study was not designed to elucidate the mechanism of the interaction between piperaquine and nevirapine, studies in future should aim to explore and define these mechanisms, which could include competitive inhibition of metabolic enzymes (26) or variations in the availability of proteins to transport drugs (27).

Evidence on the interaction between piperaquine and LPV/r-based ART is sparse. In step 1, we found an expected but nonsignificant tendency toward higher piperaquine exposure (AUC_0–28 days_) in the LPV/r ART group than in the ART-naive group but were unable to further evaluate this finding with a larger sample size in step 2 due to a limited number of study participants on this second-line ART regimen during the study period. Since LPV/r is increasingly being used as a second-line antiretroviral therapy in settings where malaria and HIV infection are endemic, its impact on piperaquine's PK profile needs to be further studied.

Previous studies found that lower day 7 plasma piperaquine concentrations are associated with recurrent malaria (28, 29). The lack of significant evidence of a difference in day 7 piperaquine concentrations between the EFV or NVP-ART group and the ART-naive group could be due to the small number of participants that had day 7 piperaquine concentrations that were above the lower limit of quantification of our assay, which may not have been able to detect low piperaquine concentrations. As efavirenz has been shown to also lower day 7 piperaquine concentrations in pregnant women (21), future studies should further explore this in HIV-infected, nonpregnant adults.

We found no major differences in the incidence of neutropenia, transaminitis, and QTc prolongation across the various ART groups, which is reassuring. However, these results need to be interpreted with caution, since this study was not powered to detect differences in safety endpoints.

The concomitant intake of piperaquine with food has previously been shown to increase the bioavailability of piperaquine (30). A lack of food restriction in step 1, including the intake of fat-containing food, may have resulted in the increased absorption of piperaquine in this step, with a subsequent higher AUC_0–28 days_ in step 1 than in step 2. Although assessing dose proportionality was not the primary aim of this study, dose normalization of the AUC_0–28 days_ (adjusting for the effect of the total administered dose) showed that there was evidence of dose proportionality between the two steps. The inability to detect significant differences in PK parameters, including dose proportionality between steps 1 and 2, may be due to the use of the parallel-group design, which is more prone to the effects of interindividual anthropometric and genetic variations than a crossover design. Thus, other covariates, such as genetic polymorphisms in CYP450 isoenzymes, may have contributed to the very wide interquartile ranges of PQ PK parameters observed within each study group and between the two steps. However, because of our study sample size, our study was unlikely to have missed large (>2-fold), clinically important differences in AUC across the study arms. Nevertheless, future studies need to assess the effect of genetic polymorphisms in CYP450 isoenzymes on the pharmacokinetics of piperaquine and quantify any changes in plasma ART levels when ART is coadministered with antimalarial drugs.

In our study, we did not assess the impact of ART on the PK profile of the faster-acting and potent partner drug of piperaquine, dihydroartemisinin. In future, studies should aim to examine any potential impact of ART on the PK profile of dihydroartemisinin and evaluate its association with parasite clearance rates among malaria-HIV-coinfected individuals.

In conclusion, this study found that although it was generally well tolerated, coadministration of piperaquine and an efavirenz-based ART regimen significantly lowered piperaquine's exposure among nonmalaria HIV-infected adults compared to that in an ART-naive subgroup. There were no major variations in piperaquine's exposure among the ART-naive participants and participants on nevirapine- and ritonavir-boosted lopinavir-based ART. The pharmacodynamic implications of these findings need to be evaluated in programmatic settings, especially in malaria-infected individuals.

## MATERIALS AND METHODS

### Study design and population.

We conducted an open-label, sequential-group, PK trial from August 2010 to March 2013 at Queen Elizabeth Central Hospital, Blantyre, Malawi. The study was implemented in the following two steps.

In step 1 (WHO International Clinical Trials Registry Platform ID number PACTR2010030001871293), we administered half adult doses of DHA-PQ (Euratesim; Sigma Tau) to the following groups of malaria-negative research participants (*n* = 6/group): (i) an antiretroviral-naive HIV^+^ (control) group, (ii) HIV^+^ individuals on NVP-based ART, (iii) HIV^+^ individuals on EFV-based ART, and (iv) HIV^+^ individuals on LPV/r-based ART.

DHA-PQ was administered orally at 0, 24, and 48 h (once daily for 3 days). One tablet (each containing DHA and PQ at 40 mg and 320 mg, respectively) was administered orally for study participants weighing <60 kg, and 1.5 tablets were administered to participants weighing ≥60 kg. Food intake, including fat-containing food, was not restricted. This step served as a safety evaluation step for the drug interaction studies, checking for unexpected clinical toxicities or interactions.

In step 2 (WHO International Clinical Trials Registry Platform ID number PACTR2010030001971409), after review and consideration of the step 1 data by an independent data safety monitoring board (DSMB), a full standard dose DHA-PQ (3 tablets to study participants weighing <60 kg and 4 tablets to those weighing ≥60 kg) was administered to 40 adults in the following groups of malaria-negative research participants (different from those enrolled in step 1): (i) an antiretroviral-naive HIV^+^ (control) group, (ii) HIV^+^ individuals on NVP-based ART, and (iii) HIV^+^ individuals on EFV-based ART.

DHA-PQ was administered at 0, 24, and 48 h (once daily for 3 days). The group of HIV^+^ individuals on LPV/r-based ART was dropped owing to the limited number of participants on this regimen available for recruitment into the study. Unlike in step 1, DHA-PQ was administered with water only in step 2; no food was given to study participants taking DHA-PQ within a period of 3 h before and 3 h after administering the drug, on the basis of a new recommendation from the drug manufacturer, Sigma Tau. In the ART arms, the first dose of DHA-PQ was timed to coincide with the next scheduled dose of the ART.

The study populations for step 1 and step 2 were HIV^+^ male and nonpregnant female participants aged ≥18 years residing in Blantyre, Malawi, or the neighboring districts of Thyolo and Chiradzulu. Individuals on ART were eligible to participate if they had been on an NVP-, EFV-, or LPV/r-based ART for ≥6 months and had a CD4 cell count of ≥250 cells/mm^3^. At the beginning of the study, HIV^+^ antiretroviral-naive individuals were eligible for ART if they had a CD4 cell count of ≥250/mm^3^, but this cutoff point was increased to ≥350/mm^3^ when the WHO criteria for ART initiation changed in July 2011. Other inclusion criteria were a body weight of ≥40 kg and a willingness to be admitted to the hospital for 3 days, to remain within the study sites, and to be contacted at home or by phone during the course of the study.

We excluded participants who had body mass index of ≤18.5 kg/m^2^; had a hemoglobin concentration of <8.5 g/dl; reported the use of any antimalarial drugs within the preceding 4 weeks; reported hypersensitivity to any of the ACTs; were taking other drugs which are known inhibitors or inducers of P450 enzymes or P-glycoprotein (except co-trimoxazole prophylaxis); had a history of regular intake of alcohol (more than twice a week), tobacco (>3 times/week), or any use of illicit drugs; had a history or evidence of preexisting liver, kidney, or heart disease, including conductive abnormalities on electrocardiographs (QTc interval, >450 ms in men and >470 ms in women); and had clinical and/or laboratory evidence of P. falciparum malaria, hepatitis B, pneumonia, tuberculosis, or bacteremia or laboratory evidence of potentially life-threatening white blood cell disorders, such as an absolute neutrophil count of <0.500 × 10^9^/liter, an absolute lymphocyte count of <0.35 × 10^9^/liter, or an absolute platelet count of <25 × 10^9^/liter. Participants who had a performance (Karnofsky) score of <80% and who were participating in any other clinical trial were also not included.

In step 1, the sample size was 6 in each of the DHA-PQ-ART and control (ART-naive) groups. This sample size was based on standard practice in early PK studies of antimalarial drugs, which aims to safeguard the safety of study subjects and minimize the number of subjects who may potentially be exposed to harmful drug levels. In step 2, a sample size of 15 per group in the DHA-PQ-ART groups and 10 in the ART-naive group was required. This was calculated to detect a 2-fold increase in the PQ AUC in any of the DHA-PQ-ART groups compared with that in the ART-naive group, assuming a mean PQ AUC of 19.4 μg · h/ml (standard deviation, 15.0 μg · h/ml) (17) in the ART-naive group, with the power set at 90% and the level of significance set at 5%.

### Ethics and data collection procedures.

The design and timing of the trial procedures were approved by the College of Medicine Research Ethics Committee (COMREC) in Blantyre, Malawi. The study conformed to the principles of the International Conference on Harmonization on Good Clinical Practice. Research nurses and clinicians sought written informed consent from individuals to perform screening procedures, including physical, medical, and anthropometric assessments, electrocardiographs (ECGs), and blood tests to detect blood-borne infections and hematological, renal, or hepatic abnormalities. The results from the screening procedures were available within 7 days of screening. On the basis of these results, potential study participants were informed of their eligibility to participate in the study. Thereafter, research nurses or clinicians sought written informed consent from eligible subjects to participate in the study.

### Pre-DHA-PQ dosing procedures.

Consenting study participants were reassessed by research nurses or clinicians to determine whether they still met all eligibility criteria through a repeat history taking and physical examination. Eligible participants were admitted in hospital, and an indwelling cannula was inserted into a vein before their scheduled dose of ART and the first dose of the ACT. At approximately 1 h before the scheduled time of ART and ACT dosing, blood samples were collected for hematological, renal, and liver function tests and also a random glucose test.

### Blood sample collection and processing.

While the participant was hospitalized, blood samples for pharmacokinetic (PK) assays were collected in heparin Vacutainer tubes before treatment and at the following posttreatment times: 0.25, 0.5, 1, 1.5, 2, 3, 4, 5, 6, 8, 12, 24, 36, 48, 60, and 72 h. After discharge, the blood samples were taken at the following times; 4, 5, 6, 7, 14, 21, and 28 days. Immediately after collection, the blood samples were spun in a refrigerated centrifuge, and the separated plasma samples were temporarily frozen in liquid nitrogen before they were transferred to a −80°C freezer until high-performance liquid chromatography (HPLC) analyses.

### Safety assessments.

After the first dose of DHA-PQ, blood samples to detect hematological, renal, and liver function abnormalities were collected at the following times; 12, 48, and 72 h and 7, 14, 21, and 28 days. In addition, 12-lead ECGs were performed before dosing, at 5 h after the first dose, and at 5 h after the last dose to assess Fridericia's-corrected QTc interval (31). The study focused on treatment-emergent adverse events (TEAEs), defined as clinical or subclinical abnormalities which were absent before dosing with DHA-PQ but emerged postdosing or those which were present before dosing with DHA-PQ but worsened postdosing. The severity of the adverse events was graded using the Division of AIDS criteria (32), while seriousness was defined according to the standard definition.

### Pharmacokinetic assays.

Plasma samples were analyzed for PQ levels at the Malawi-Liverpool-Wellcome Trust Clinical Research Programme in Blantyre, Malawi, using a validated HPLC-UV assay adopted and transferred to Malawi from the Liverpool School of Tropical Medicine. The PK laboratory in Blantyre participated in the World Wide Antimalarial Resistance Network's external quality assurance program (33). Briefly, PQ and the internal standard (chloroquine) were recovered from plasma using diethyl-*tert*-butyl ether. The supernatant was evaporated to dryness in a vacuum concentrator at 25°C. The residue was redissolved in 200 μl of the reconstitution solvent acetonitrile-phosphate buffer (5:95, pH 2.5), and 75 μl was injected into the chromatograph (Agilent 1100). Quantitation of the drugs was achieved by reverse-phase HPLC. The optimum detection wavelength for each drug was 345 nm. The lower limit of quantitation (LLOQ) of the piperaquine HPLC-UV assay was 0.025 μg/ml with a coefficient of variation of <10%. Reconstituted plasma sample extracts were run in batches comprising all samples collected from each of any two study participants. Each batch run included a blank plasma extract, two sets of 8-concentration-level calibration standards, and quality controls (QC) at three concentrations: low, medium, and high (0.025, 1.5, and 3.0 μg/ml, respectively, for PQ). For a batch assay to pass, the measured concentrations of at least 67% of the QC samples had to be within ±20% of their nominal value and at least one QC sample had to be acceptable at the LLOQ. The mean interassay precision for the low-, medium-, and high-concentration QCs was 7%, 12%, and 10%, respectively. In addition, 75% of each calibration curve's concentrations had to lie within ±20% and ±15% of the nominal concentration at the LLOQ or all other concentrations, respectively.

### Pharmacokinetic and safety data analyses.

Plasma concentrations of piperaquine were analyzed using noncompartmental pharmacokinetic analysis (NCA), employing the trapezoidal rule with cubic splines. Observed piperaquine concentrations below the lower limit of quantification (<LLOQ) were treated as missing data, except for the predose concentration, which was imputed to 0 if it was below the LLOQ. For each study participant, the following PK parameters were computed: AUC_0–28 days_, maximum concentration (*C*_max_), the time to the maximum concentration (*t*_max_), and the terminal elimination half-life (*t*_1/2_). We used STATA (version 15.0) software for the NCA and to compare log-transformed PK parameters. Geometric mean ratios with 90% confidence intervals are presented. To test for significant differences in PK parameters between each ACT/ART group and the ART-naive group, parametric evaluation of the log-transformed PK parameters was done using analysis of variance (ANOVA) (α = 0.1). Fisher's exact test was used to compare the proportions of participants across the study groups with day 7 concentrations that were above a value known to predict the treatment response by day 28 and to compare the safety parameters across the different ACT/ART groups to those for the ART-naive group. Data summaries and graphics were all performed in STATA (version 15.0).
